# Asteatotic eczema, a cutaneous manifestation of Hodgkin lymphoma in older patients

**DOI:** 10.1002/jha2.910

**Published:** 2024-06-26

**Authors:** Ariane Le Clainche Compagnie, Charlotte Degoutte, Barbara Papouin, Claire Lamaison, Romain Gounot, Saskia Ingen‐Housz‐Oro

**Affiliations:** ^1^ Hopital Henri Mondor, Dermatology Créteil France; ^2^ Hopital Henri Mondor, Haematology Créteil France; ^3^ Hopital Henri Mondor, Anatomopathology Créteil France

**Keywords:** asteatotic eczema, elderly, hodgin lymphoma

1

An 82‐year‐old man was referred for a 13 kg weight loss and an erythroderma evolving for 2 months. Clinical examination revealed an asteatotic eczema (AE) predominant on the trunk (Figure 1A) associated with palmoplantar keratoderma and supracentimetric cervical polyadenopathy. Biologically, the patient presented with 11 g/dL nonregenerative normocytic anemia, neutrophilic polynucleosis of 12.4 G/L, lymphopenia of 0.4 G/L, and hypoalbuminemia of 21 g/L. Imaging (computed tomography [CT] scan and positron emission tomography (PET)‐CT) confirmed supradiaphragmatic hypermetabolic polyadenopathy. Skin biopsy showed ichthyosiform dermatosis suggestive of a paraneoplastic process (Figure 1C). Node biopsy confirmed the diagnosis of classic Hodgkin lymphoma (Figure 1D). The patient was treated with brentuximab vedotin alone for the first two perfusions which permitted AE clinical complete remission (CR) (no PET‐CT reevaluation was available) and then combined with pembrolizumab for treatment intensification which was effective on both the lymphoma and the dermatosis, and which permitted a CR after four cycles. Treatment was stopped after four doses of brentuximab vedotin and two doses of pembrolizumab because of CR and immunomediated toxicities (nephropathy and pancreatitis). He is still in CR after 10 months of follow‐up.

**FIGURE 1:**
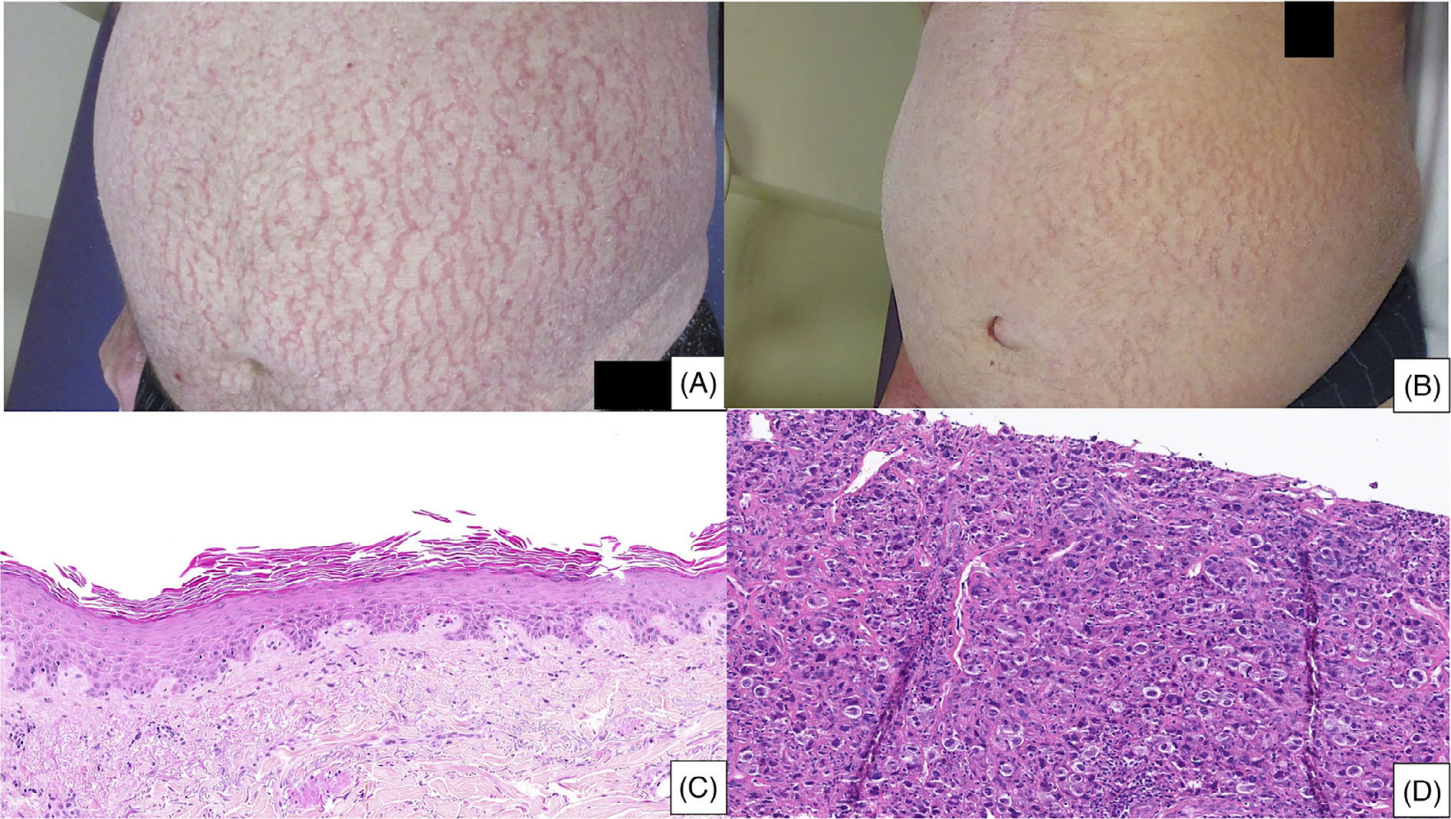
(A) Asteatotic eczema aspect on the first patient's trunk: deep inflammatory cracks. (B) Asteatotic eczema aspect on the second patient's trunk. (C) Patient's 1 skin biopsy: acanthosis hyperplastic epidermis with lamellar parakeratosis with dissociated corneocytes, spongiosis, no inflammatory cell infiltrate. (D) Patient's 1 lymph node biopsy: lymphoproliferation of atypically large, nucleated cells CD30+, ALK1‐, PAX5+, CD15+, CD4+, BOB1+, perforin+, EMA+, STAT3+, CD20‐, CD19‐, CD79A‐, OCT2‐, CD22‐, CD2‐, CD3‐, CD5‐, CD7‐, CD8‐, granzyme B‐, and TIA1‐; compatible with Hodgkin lymphoma.

A 78‐year‐old man was hospitalized for a diffuse AE associated with palmoplantar keratoderma (Figure 1B). A single firm and painless right inguinal adenopathy measuring 3 cm was found at clinical examination. The CT scan showed primitive and external right iliac adenopathies. Node biopsy was consistent with Epstein‐Barr virus‐negative classic Hodgkin lymphoma. The patient died suddenly of unknown cause before beginning the treatment.

AE, first described in 1907 by Bocq, is a particular form of xerosis affecting older patients, which results from a depletion of the secretion of the sebaceous and sweat glands [1]. It may be present in healthy individuals, but certain phenotypic features such as deep, inflammatory cracks with trunk predominance should outweigh possible underlying neoplasia. The association between AE and hemopathies is already described, especially in non‐Hodgkin lymphoma [2]. The association with classic Hodgkin lymphoma is rarer. Skin manifestations are present in 17%–53% of patients with classic Hodgkin lymphoma, mainly characterized by non‐specific lesions, and can be the disease's gateway [3]. The most frequent skin manifestation is pruritus (20% of cases). Other manifestations include polymorphic skin symptoms, such as prurigo, paraneoplastic pemphigus, erythema multiforme, erythema nodosum, and various kinds of eruptions (bullous, eczematous, and psoriatic). Clinicians should recognize AE by its presentation and be aware that it may reveal Hodgkin lymphoma, especially in older patients.

## CONFLICT OF INTEREST STATEMENT

The authors declare no conflict of interest.

## FUNDING INFORMATION

No funding has been received for this article.

## ETHICS STATEMENT

The authors have confirmed ethical approval statement is not needed for this submission.

## PATIENT CONSENT STATEMENT

We have patient consent for this plubication in patient consent statement.

## CLINICAL TRIAL REGISTRATION

The authors have confirmed clinical trial registration is not needed for this submission.

## Data Availability

Data sharing is not applicable to this article as no new data were created or analyzed in this study.
